# From Bilateral Periorbital Necrotic Wound to Fungal Brain Abscess: A Complicated Case of COVID-19-Associated Mucormycosis

**DOI:** 10.1155/2022/3821492

**Published:** 2022-08-14

**Authors:** Bahram Eshraghi, Nastaran-Sadat Hosseini, Rasoul Mohammadi, Seyed Hamid Reza Abtahi, Alireza Ramezani-Majd, Roya Azad, Mohsen Pourazizi

**Affiliations:** ^1^Isfahan Eye Research Center, Department of Ophthalmology, Isfahan University of Medical Sciences, Isfahan, Iran; ^2^School of Medicine, Isfahan University of Medical Sciences, Isfahan, Iran; ^3^Department of Medical Parasitology and Mycology, School of Medicine, Infectious Diseases and Tropical Medicine Research Center, Isfahan University of Medical Sciences, Isfahan, Iran; ^4^Department of Otolaryngology, Isfahan University of Medical Sciences, Isfahan, Iran; ^5^Department of Radiology, Faculty of Medicine, Isfahan University of Medical Sciences, Isfahan, Iran

## Abstract

COVID-19-associated mucormycosis (CAM) is categorized as rhinocerebral-orbital (RCOM), pulmonary, gastrointestinal, cutaneous, and disseminated mucormycosis. An alarming surge in morbidity and mortality attributed to mucormycosis concurrent with coronavirus disease 2019 (COVID-19) has emerged as a cause for concern during the current outbreak of COVID-19. The global incidence of CAM has been attributed to environmental, host, and iatrogenic factors. Further, *Mucorales* interacting with epithelial cells followed by endothelium invasion are pivotal in developing mucormycosis in patients with COVID-19. In essence, CAM is an emerging condition that requires increased vigilance in all COVID-19 patients, including those who have recovered. In this case report, we describe a rare case of CAM in a 33-year-old immunocompetent man who developed bilateral periocular pain and a small area of cutaneous necrosis in both medial canthi associated with impaired vision, which progressed into a fungal brain abscess formation in the post-COVID period. Furthermore, this case aims to illustrate the potential underlying risk factors of CAM other than known risk factors, especially in immunocompetent individuals.

## 1. Introduction

Coronavirus disease 2019 (COVID-19) pandemic has led to a surge in invasive fungal infections, particularly among critically ill patients [1]. Mucormycosis is an invasive fungal infection caused by order *Mucorales*. The causal agents are thermotolerant, rapid-growing fungi belonging to the order *Mucorales* [2]. The capacity of these fungi to infect immunocompetent hosts is uncommon, despite their widespread occurrence in the environment. Patients, in the majority of instances, have underlying health issues [2]. Since the high prevalence is temporally associated with COVID-19, the entity is called COVID-19-associated mucormycosis (CAM). Although an exact causative relationship between COVID-19 and mucormycosis has not been proved, various factors were linked to the development of mucormycosis in COVID-19 patients, including (a) an unexpected increase in the circulating levels of proinflammatory cytokines, particularly interleukin-6 (IL-6) and tumor necrosis factor-*α* (TNF-*α*), (b) acute hyperglycemic state, (c) metabolic acidosis, (d) inappropriate immune system response, (e) specific environmental conditions contributed to high spore burden, (f) altered iron metabolism, (g) endotheliitis and microvasculopathy, (h) upregulation of GRP-78, (i) iatrogenic factors (e.g., adverse events associated with corticosteroids, IL-6 inhibitors, etc.), and (j) comorbid conditions, such as diabetes mellitus and obesity [1, 3–6].

Rhinocerebral-orbital mucormycosis (RCOM) is the most frequent clinical kind with the highest mortality rate, followed by pulmonary illness. About three-fourths of them had diabetes as predisposing comorbidity and were treated for COVID-19 with corticosteroids [3]. The involvement of brain tissue may manifest as altered awareness, headache, unsteady gait, stroke, or seizures [7, 8]. Once *Mucorales* first established in the nasal cavity and destroy the adjacent bone and soft tissues in this area, it proceeds to the maxillary and ethmoid sinuses, orbits, cavernous sinuses, meninges, and finally, the brain in terms of its angioinvasive propensity. Mucormycosis can spread to the intracranial cavity via the perivascular channels from the paranasal sinuses or via the cribriform plate into the anterior cranial fossa [9].

Hence, we report a rare case of a complicated case of CAM with a catastrophic course with a fungal brain abscess in a 33-year-old male with no remarkable past medical history.

## 2. Case Presentation

**Figure 1:**
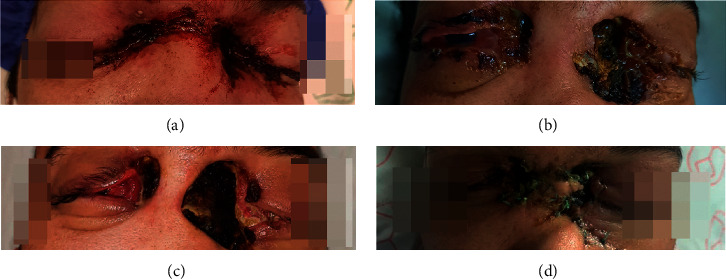
The clinical course of COVID-19-associated mucormycosis: (a) cutaneous manifestation of bilateral periorbital mucormycosis. The small area of skin necrosis on bilateral sides; (b) the necrotic epidermal lesion with ill-defined raised margins and a necrotic base significantly worsened and expanded from the medial canthi, eyelids, and periorbital area; (c) progressive cutaneous necrosis associated with the midfacial defect; (d) repaired cutaneous lesions after medical and surgical treatment.

A 33-year-old previously healthy male with no history of COVID-19 vaccination was diagnosed with a positive reverse transcription-polymerase chain reaction (RT-PCR) for severe acute respiratory syndrome coronavirus-2 (SARS-CoV-2). He had no history of diabetes, immunosuppressive disorders, or cancer. After a diagnosis of moderate to severe COVID-19 pneumonia, the patient was administered remdesivir and methylprednisolone. After 10 days of hospitalization, he was released in a relatively good condition. Five days after discharge, he developed bilateral periocular pain, blurred vision, and a small area of cutaneous necrosis in both medial cantus. On examination, mild facial edema, periorbital edema, and facial paresthesia were apparent (Figure 1(a)).

Moreover, the patient reported a decrease in visual acuity. There was no evidence of a lesion inside the mouth. During the course of the study, hyperglycemia was identified (a random blood sugar of about 400). All paranasal sinuses had mucosal thickening and partial opacification, which was linked to orbital involvement with retrobulbar invasion. During the ocular evaluation, ptosis, proptosis, and chemosis, concomitant ophthalmoplegia and bilateral loss of vision were discovered (the identified visual acuity, using the semiquantitative scale, was light perception (LP) in both eyes). The patient was put on intravenous liposomal amphotericin B at 5 mg/kg/day after a clinical diagnosis of mucormycosis.

Extensive erosion of the nasal septum, bilateral middle and inferior turbinates, and anterior and severe necrosis of the posterior ethmoidal mucosal membrane and lamina papyracea were all seen during the initial endoscopic sinus surgery (ESS). The debridement of the base of the skull was conducted, as well as the complete removal of the nasal septum in the middle and posterior portions and the turbinectomy on both sides. The lamina papyracea and preorbital fat were debrided to the point of apparent necrosis.

The patient's cutaneous lesions deteriorated on the second day of his admission in terms of the disease's rapid progression (Figure 1(b)). The patient's visual acuity varied to a state of no light perception (NLP). Liposomal amphotericin B 3.5 mg/ml was administered to the patient via retrobulbar injection.

Following substantial midfacial bone erosion and skin necrosis, the second surgery was performed (Figure 1(c)). In the second surgery, intraoperative results indicated a necrotic lesion extending from the inferior border of the left orbit to both sides of the nasal bones, which was debrided. The skin in the midface area had a considerable defect, which was reconstructed using advancement flaps following maximal debridement of the bone and skin (Figure 1(d)). Patients reported persistent headaches and fever on the seventh day despite continued antifungal treatment.

A contrast-enhanced brain and orbital magnetic resonance imaging (MRI) revealed the destruction of sinuses walls and intraorbital and intracranial extension with bilateral frontal and right temporal lobe abscess formation and intraconal and extraconal fat edema and thickening and hyperintensity of extraocular muscles in both orbits, consistent with invasive fungal sinusitis.

**Figure 2:**
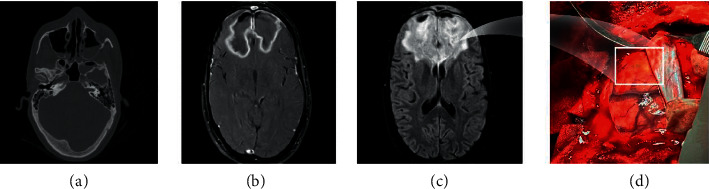
Radiologic characteristics of COVID-19-associated mucormycosis: (a) axial bone window CT scan demonstrated partial opacification of bilateral maxillary and sphenoid sinuses. (b) Axial postcontrast T1 weighted image revealed smooth ring enhancement in bilateral frontal lobes mass; (c) axial fluid-attenuated inversion recovery MRI showed a hyperintense mass containing air bubbles with perilesional vasogenic edema in bilateral frontal lobes; (d) drainage of the yellow brain abscess material using craniotomy.

The patient's condition had worsened to the point that he was confused. As a result of a mass lesion in the frontal lobe, he underwent craniotomy (Figure 2).

**Figure 3:**
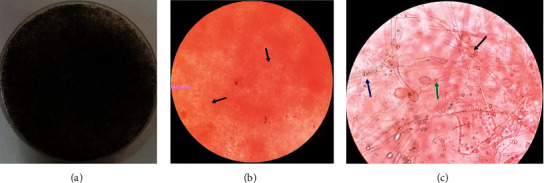
Mycology confirmation and detection of fungal elements by phenotypic methods: (a) grayish brown colony of Rhizopus spp. on Sabouraud dextrose agar with chloramphenicol; (b) direct microscopy with potassium hydroxide 10% (KOH 10%) shows broad and aseptate hyphae of Rhizopus spp. in necrotic tissue (black arrows), ×40; (c) reproductive structures of *Rhizopus* spp. sporangium (black arrow), sporangiophore (blue arrow), and sporangiospores (green arrow), ×40.

Direct examination of the biopsy of involved tissue in the fungal abscess with potassium hydroxide 10% (KOH 10%) showed aseptate hyphae in necrotic tissues compatible with mucormycosis. A three-day-old culture on Sabouraud dextrose agar (Merck, USA) with chloramphenicol revealed a greyish-brown colony of *Rhizopus* spp. and broad sporangiophores with sporangia and round sporangiospores under the microscope. Biopsy reports from the affected sinuses were in agreement with this conclusion (Figure 3).

After a few weeks, the patient was released with bilateral blindness. Despite this, there was no evidence of disease recurrence during the four-month follow-up.

## 3. Discussion

It is extremely rare for an immunocompetent patient to present with bilateral orbital mucormycosis accompanied by a brain abscess in the post-COVID-19 phase. The clinical importance of our report was its bilateral presentation and CNS progress, so it is important to consider cerebral CAM in any patient with an altered level of consciousness in the setting of CAM.

In general, mucormycosis is an opportunistic and life-threatening infection usually associated with certain predisposing conditions, such as uncontrolled diabetes, use of corticosteroids, immunosuppressive therapy, immunodeficiency state, malignancies, transplantation, iron overload, intravenous drug use, chronic alcoholism, and malnutrition [10]. In particular, diabetes mellitus, particularly with ketoacidosis, is the most commonly reported risk factor in non-COVID-19 mucormycosis, for 54–76% of such cases in India, Iran, and Mexico [11].

During the COVID-19 pandemic, the clinicoepidemiological pattern of mucormycosis changed, with typical risk factors such as diabetes, transplantation, and hematological malignancies being absent. The development of CAM can probably be attributed to acute hyperglycemia. Thus, using glucocorticoids in mild COVID-19 cases (without hypoxemia) or using higher doses of glucocorticoids should be avoided [12].

*Mucorales* spores are widely distributed in soil, decaying organic material, and animal feces. Our patient's susceptibility to mucormycosis infection and germination and multiplication of the fungus spores may have been increased as a result of his history of smoking Anbarnesa, a traditional Iranian medicine, or using unclean humidifiers and oxygen delivery masks [3, 6, 13]. The inhalation of Anbarnesa smoke is used to cure a variety of illnesses, including viral infections. Anbarnesa has a considerable impact on the rapid healing of various types of ulcers, notably burn wounds. It has the most potent antioxidant property in higher doses [14].

In our patient, one of the primary signs and symptoms of CAM was secondary cutaneous mucormycosis in the periorbital area. Secondary cutaneous mucormycosis usually develops as a result of disseminated or rhinocerebral infection. Tissue infarction may occur quickly, resulting in the appearance of a black eschar with central ulceration [15]. During infection, the mentioned patient develops progressively spreading cutaneous necrosis. Although the relationship between facial cutaneous necrosis and poor prognosis was statistically insignificant, it appears to be clinically meaningful [15, 16].

Due to the continuous spread of the paranasal sinuses and orbits, CNS involvement is regarded as a significant and unusual consequence of CAM [1, 7]. Possible pathway for brain involvement during ROCM includes (a) nasopharyngeal mucosa penetration and spreading to the brain via blood or lymphatic vessels, (b) proliferation in the internal elastic lamina, entering the endothelium and subsequently obstructing the cerebral vascular lumen, (c) hematogenous transmission via perforating branches of the middle cerebral artery, and finally, (d) host innate immune cells impairment and decrease in antifungal drugs access to Mucorales in terms of the hemorrhagic necrosis and infarction of the brain [17, 18].

Because antifungals cannot reach necrotic tissues, vigorous surgical debridement of necrotic tissues should be explored as soon as a diagnosis of any type of CAM is suspected. Debridement is often disfiguring in instances of rhinocerebral infection, requiring the removal of the palate, nasal cartilage, and orbit. Recent evidence suggests that endoscopic debridement can be accomplished with minimal tissue loss [19, 20]. The treatment of choice for initial antifungal therapy in CAM is intravenous amphotericin B. Patients who do not respond to or cannot tolerate amphotericin B may even receive posaconazole or isavuconazole as salvage or alternative therapy [19]. There are no randomized studies to manage the neurological complications associated with CAM; most of the recommendations are essentially based on expert's opinions.

Decompressive craniectomy may be done in space-occupying lesions to reduce intracranial pressure (ICP) [19]. The management of septic cerebral venous disease, including cavernous sinus thrombosis, in patients with CAM, is challenging. On the one hand, thrombosis formation in CNS is associated with known CNS complications, and on the other hand, thrombosis can prevent the spread of infection, there is a risk of intracranial haemorrhage due to the angioinvasive nature of the fungus. As a result, no firm conclusions can be drawn about the use of systemic anticoagulants in the management of septic cerebral venous thrombosis in the context of CAM [8, 21]. Routine use of antibiotics and prophylactic use of antiepileptic medication are not recommended [19].

## 4. Conclusion

Mucormycosis should be included in the differential diagnosis when there is no obvious explanation for a worsening clinical course in COVID-19 area. Clinicians should be aware of the potential factors related to CAM and the critical role of optimal glycemic control. A multidisciplinary team approach to addressing fungal infections is essential since it involves early identification, surgical removal of contaminated tissue, optimum antifungal medication, and follow-up care. They should boost their clinical knowledge of the mucocutaneous, ocular, and central nervous system involvement of CAM and educate their patients about these warning signs to provide prompt diagnosis and timely medicinal or surgical management to have a promising outcome.

## Data Availability

Data are available on request.
